# A novel structural maintenance of chromosomes (SMC)-related protein family specific to Archaea

**DOI:** 10.3389/fmicb.2022.913088

**Published:** 2022-08-05

**Authors:** Mari Yoshinaga, Takuro Nakayama, Yuji Inagaki

**Affiliations:** ^1^Graduate School of Science and Technology, University of Tsukuba, Tsukuba, Japan; ^2^Center for Computational Sciences, University of Tsukuba, Tsukuba, Japan

**Keywords:** SMC superfamily, ATPases, Archaea, coalescin, Sph, Arcadin-4

## Abstract

The ATPases belonging to the structural maintenance of chromosomes (SMC) superfamily are involved in the maintenance of chromosome organization and dynamics, as well as DNA repair. The major proteins in this superfamily recognized to date are either conserved among the three domains of Life (i.e., SMC and Rad50) or specific to Bacteria (i.e., RecF, RecN, and MukB). In Archaea, no protein related to SMC (SMC-related protein) with a broad taxonomic distribution has been reported. Nevertheless, two SMC-related proteins, namely coalescin and Sph, have been identified in crenarchaea *Sulfolobus* spp. and the euryarchaeon *Halobacterium salinarum*, respectively, hinting that the diversity of SMC-related proteins has been overlooked in Archaea. In this study, we report a novel SMC-related protein that is distributed among broad archaeal lineages and termed “Archaea-specific SMC-related proteins” or “ASRPs.” We further demonstrate that the ASRP family encloses both coalescin and Sph but the two proteins represent only a tip of the diversity of this family.

## Introduction

Structural maintenance of chromosomes (SMC) proteins are ATPases that participate in maintaining the integrity of chromosome structure and have been found in all the domains of Life, indicating their significance for cellular viability (Losada and Hirano, 2005). The SMC proteins identified so far can be split into two “clusters” in phylogenetic analyses–one containing SMC1-4 in Eukaryota, bacterial SMC, and the “canonical” version of archaeal SMC, and the other containing SMC5 and SMC6 in Eukaryota and the “SMC5/6-related” version of archaeal SMC (Yoshinaga and Inagaki, 2021). As ATPases, SMC proteins have a set of conserved sequence motifs that are required for ATP binding and hydrolysis (i.e., Walker A, Walker B, and signature motifs) but these motifs are not in close proximity to one another in their primary structures (Losada and Hirano, 2005). Walker A motif at the N-terminus is separated from Walker B and signature motifs at the C-terminus by the amino acid sequence that constitutes coiled-coil and “hinge” in the tertiary structure.

The unique feature in distribution of the ATP binding motifs in SMC described above has been found in other ATPases, namely Rad50, RecN, RecF, and MukB, and these proteins, together with SMC, as a whole have been referred to as the “SMC superfamily” (Cobbe and Heck, 2000, 2004). Rad50, which is involved in DNA repair, is ubiquitous in the three domains of Life (the Rad50 homologs in Bacteria are termed as SbcC) (Kinoshita et al., 2009). On the other hand, RecN, RecF, and MukB are Bacteria-specific (Cobbe and Heck, 2004). Both RecN and RecF are involved in DNA repair and are conserved in diverse lineages of Bacteria (Hegde et al., 1996; Keyamura et al., 2013). In *Escherichia coli*, SMC is absent but instead, MukB was experimentally shown to fulfill the SMC function (Niki et al., 1991). So far, MukB has been found mainly in γ-proteobacteria including *E. coli* (Cobbe and Heck, 2004). In contrast to Eukaryota and Bacteria, it remains unclear whether the diversity of SMC-related proteins in Archaea is sufficiently understood. Ruepp et al. (1997) identified a 71-kDa protein, Hp71, in the euryarchaeon *Halobacterium salinarum* and its predicted structure resembles SMCs. The over expression of Hp71 in euryarchaeal cells (i.e., *H. salinarum* and *Haloferax volcanii*) altered the cell morphology, implying that this protein is involved in a cytoskeletal-like structures (Ruepp et al., 1997). Later, Hp71 appeared to correspond to one of the two “SMC like proteins in *H. salinarum*” or “Sph” (Herrmann and Soppa, 2002). Although Sph was proposed to be involved in DNA repair (Herrmann and Soppa, 2002), its precise function has yet to be clarified. Another SMC-related protein, Archadin-4, was found in Thermoproteales archaea (e.g., *Pyrobaculum calidifontis*) and experimentally shown to associate with cytoskeleton (Ettema et al., 2011). More recently, an SMC-related protein termed coalescin or ClsN was found in crenarchaea *Sulfolobus acidocaldarius* and *Sulfolobus islandicus* (Takemata et al., 2019). *Sulfolobus* spp. possess no SMC (Kamada and Barillà, 2018) but ClsN and a series of experiments indicated that ClsN is responsible for discriminating the portion of the *Sulfolobus* chromosomes with low transcriptional activity from that carrying the transcriptionally active genes (Takemata et al., 2019). Importantly, Takemata et al. (2019) hinted at the potential ClsN homologs in other archaea, although no detail of these sequences was provided. Likewise, the distribution of Sph in Archaea has not been addressed in any previously published works.

To grasp the diversity of the SMC superfamily in Archaea, we systematically surveyed SMC-related proteins in the archaeal genomes/metagenomes deposited in the GenBank database in this study. We here document a previously overlooked group of archaeal SMC-related proteins, which are distinct from any of previously known SMC, Rad50, or Bacteria-specific SMC-related proteins but enclose both ClsN and Sph. The novel archaeal proteins identified here are termed “Archaea-specific SMC-related proteins” or “ASRPs.” The diversity and function of ASRPs as a whole is unlikely represented by ClsN or Sph, as the two previously known ASRPs distribute in the restricted lineages in Archaea, namely Sulfolobales and Halobacteria, respectively.

## Materials and methods

### Sequences related to *Sulfolobus islandicus* ClsN, and *Halobacterium salinarum* Sph1 and Sph2

Henceforth here, we designate *S. islandicus* ClsN (ADX84150.1) as “*Sis*ClsN.” Likewise, *H. salinarum* Sph1 and Sph2 (O07116 and Q9HHY2) are designated as “*Hsa*Sph1” and “*Hsa*Sph2,” respectively.

We searched for ClsN-related proteins in the GenBank nr database by using BLASTP (taxid = 2,157; max_target_seq = 50,000) with *Sis*ClsN as the query. 459 sequences matched with the query with *E*-values smaller than 10^–5^ were retrieved as the “ClsN candidates.” The BLASTP search was repeated twice by replacing the query from *Sis*ClsN to *Hsa*Sph1 and *Hsa*Sph2. 1687 sequences were retrieved as the “Sph1/Sph2 candidates,” which matched one of the two Sph proteins with *E*-values smaller than 10^–5^. The ClsN and Sph1/Sph2 candidates were pooled together and subjected to CD-HIT (Fu et al., 2012) with the -c 0.5 or 0.4 option to reduce the redundancy at the amino acid sequence level. Finally, we selected 111 archaeal sequences for the preliminary phylogenetic analysis (see below) as the “ClsN/Sph candidates.”

In our previous work, we prepared and analyzed a phylogenetic alignment of SMC and Rad50 amino acid sequences sampled from Eukaryota, Archaea, and Bacteria (Yoshinaga and Inagaki, 2021). We generated a smaller alignment of 20 archaeal Rad50, and 80 archaeal SMC from the pre-existing alignment. Then, the amino acid sequences of the 111 ClsN/Sph candidates, as well as *Sis*ClsN, *Hsa*Sph1, and *Hsa*Sph2, were added to the archaeal SMC/Rad50 alignment by MAFFT v7.453 (Katoh, 2002) with the L-INS-I and –merge options. 13 out of the 111 ClsN/Sph candidates were discarded due to their highly diverged sequence natures or lack of Walker A motif at the N-terminus or Waker B and signature motifs at the C-terminus. Prior to the phylogenetic analyses described below, we refined the alignment by the manual exclusion of ambiguously aligned positions, coupled with trimming of gap-containing positions by trimAI v1.2 (Capella-Gutierrez et al., 2009) with -gt 0.8 option. The resultant alignment comprised *Sis*ClsN, *Hsa*Sph1, *Hsa*Sph2, 98 full-length ClsN/Sph candidates, 59 canonical and 21 SMC5/6-related SMC in Archaea, and 20 archaeal Rad50 sequences with 222 unambiguously aligned amino acid positions and was subjected to the maximum-likelihood (ML) phylogenetic analysis with the LG + Γ + F + I model by using IQ-TREE version 2.1.2 (Nguyen et al., 2015) (the substitution model selected by IQ-TREE). The statistical support for the bipartitions in the ML tree was calculated by the ultrafast bootstrap approximation implemented by IQ-TREE (1,000 replicates). 15 sequences out of the 98 ClsN/Sph candidates showed the apparent phylogenetic affinities to SMC or Rad50, and the rest of the candidates (80 sequences), together with *Sis*ClsN, *Hsa*Sph1, and *Hsa*Sph2, formed a clade supported by an ultrafast bootstrap support value (UFBP) of 99%. Thus, we regard the 80 archaeal sequences as the close relatives of *Sis*ClsN, *Hsa*Sph1, and *Hsa*Sph2.

We repeated the BLAST search described above and this time against the bacterial sequences deposited in the GenBank nr database (taxid = 2; max_target_seq = 50,000). The details of the second search were the same as those of the first search (see above). 15 bacterial sequences matched with the three queries with *E*-values smaller than 10^–5^. After the inspection of the conserved motifs at the N- and C-termini and preliminary phylogenetic analysis (Supplementary Figure 1), only three out of the 15 bacterial sequences were retained as ClsN/Sph candidates. The bacterial ClsN/Sph candidates were identified in the marine sediment metagenomes of (i) *Candidatus* Cloacimonas sp. 4484_209 (OQX55830.1) and (ii) *Candidatus* Omnitrophica bacterium (RKY43431.1), and the marine metagenome of (iii) Dehalococcoidia bacterium (MBC8512469.1). In each of the three metagenome assemblies described above, (i) the genes surrounding the bacterial ClsN/Sph candidates and (ii) their origins deduced from the BLASTP searches against RefSeq Selected proteins are summarized in Supplementary Figure 2.

### Phylogenetic analyses

From the 83 archaeal sequences related to ClsN/Sph, we selected 44 sequences that represent the diversity of the clade containing *SisClsN*, *Hsa*Sph1, and *HsaSph2*. Then, the amino acid sequences of *Sis*ClsN, *Hsa*Sph1, *Hsa*Sph2, and the 41 archaea proteins related to ClsN/Sph (see above) were aligned with (i) 30 sequences that represent the six SMC subfamilies in Eukaryota, (ii) 13 sequences of the canonical SMC in Archaea, (iii) 15 sequences of “SMC5/6-related SMC” in Archaea, (iv) 17 SMC sequences in Bacteria, (v) 15 MukB sequences, (vi) 17 RecN sequences, (vii) 10 RecF sequences, (viii) 15 sequences of Rad50 in Archaea, and (ix) 8 sequences of Arcadin-4. We omitted the Rad50 orthologs in Eukaryota and Bacteria, as they are extremely rapidly evolving (Yoshinaga and Inagaki, 2021) and potentially introduce severe artifacts in tree reconstruction (e.g., long-branch attraction or LBA). All of the SMC and Rad50 sequences described above were the subset of the sequences analyzed in our previous work (Yoshinaga and Inagaki, 2021) and thus pre-aligned. *Sis*ClsN, *Hsa*Sph1, *Hsa*Sph2, and their closely related sequences were also aligned for the preliminary phylogenetic analysis (see above). The full-length MukB, RecN, RecF, and Arcadin-4 sequences were retrieved from the GenBank database and aligned individually by MAFTT v7.453 (Katoh, 2002) with the L-INS-I option. The alignments described above were then merged into a large alignment by MAFTT with the –merge option. Ambiguously aligned positions and gap-containing positions were excluded as described above, leaving 184 sequences with 232 amino acid positions. The final alignment (“SMC-superfamily” alignment) was subjected to the tree reconstruction and non-parametric bootstrap analyses by using IQ-TREE v2.1.2 (Nguyen et al., 2015). The ML tree was inferred under the LG + C60 + F + Γ model, which was selected by IQ-TREE. We also conducted the ML non-parametric bootstrap analysis with the LG + C60 + F + Γ + PMSF [posterior mean site frequencies; Wang et al. (2018)] model (100 replicates) with the ML tree used as the guide tree. We repeated the ML phylogenetic analyses described above after excluding the 8 Arcadin-4 sequences from the SMC-superfamily alignment.

We generated the second alignment by removing the sequences of SMC in Eukaryota and Bacteria, Rad50, RecN, RecF, MukB, and Arcadin-4 from the SMC-superfamily alignment. The second alignment includes *Sis*ClsN, *Hsa*Sph1, *Hsa*Sph2, the 41 archaeal proteins related to ClsN/Sph, and 13 canonical and 15 “SMC5/6-related” SMC in Archaea. The second alignment was subjected to the ML and ML non-parametric bootstrap analyses as described above. We applied the LG + C60 + F + R5 model for tree reconstruction and the LG + C60 + F + R5 + PMSF model for bootstrap analysis.

We also subjected the SMC-superfamily alignment (with and without the Arcadin-4 sequences) and the second alignment to PhyloBayes v4.1 (Lartillot and Philippe, 2004, 2006; Lartillot et al., 2007) using the CAT + GTR model. For Bayesian analysis of SMC-superfamily alignment (including Arcadin-4), two Markov chain Monte Carlo (MCMC) chains were run for more than 90,000 cycles. The first 22,500 cycles were discarded as burn-in, and the consensus tree and Bayesian posterior probabilities (BPPs) were calculated from the remaining trees (the maxdiff value was 0.172805). In the same analysis after the exclusion of Arcadin-4, we ran two MCMC chains for more than 200,000 cycles and discarded the first 50,000 cycles as burn-in to calculate the consensus tree and BPPs (the maxdiff value was 0.0611113). For the second alignment, we ran two MCMC chains for more than 275,000 cycles. After the discard of the first 70,000 cycles, the consensus tree and BPPs were calculated from the remaining trees (the maxdiff value was 0.0353794).

## Results and discussion

### A novel SMC-related protein family specific to Archaea

**FIGURE 1:**
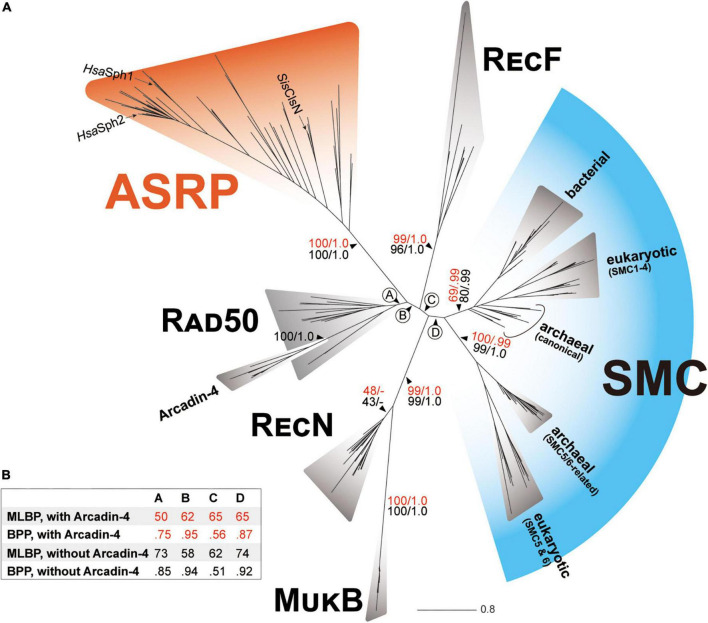
Phylogenetic relationship among SMC and SMC-related proteins. **(A)** The ML tree inferred from the SMC-superfamily alignment. There was no substantial incongruity between the ML and Bayesian trees, and thus only the ML tree is shown. For the major nodes, non-parametric ML bootstrap support values (MLBPs) and Bayesian posterior probabilities (BPPs) are presented (the support values are colored in red). We repeated both ML and Bayesian analyses on the same alignment after the exclusion of the Arcadin-4 sequences. The overall tree topology remained the same before and after the exclusion of the Arcadin-4 in the alignment and only MLBPs and BPPs calculated from the second set of the phylogenetic analyses are presented for the major nodes (shown in black). The archaeal sequences identified in this study, together with *Sulfolobus islandicus* ClsN and *Halobacterium salinarum* Sph1 and Sph2 (*Sis*ClsN, *Hsa*Sph1, and *Hsa*Sph2; the corresponding terminal branches were marked by arrows), formed a clade with full statistical support. This clade is termed “ASRP (Archaea-specific SMC-related protein).” **(B)** The MLBPs and BPPs for the nodes labeled A–D. For each of the four nodes, we summarized the MLBPs and BPPs calculated from the ML/Bayesian analyses in which the Arcadin-4 sequences were included and excluded.

In the phylogenetic tree inferred from the SMC-superfamily alignment, the SMC and RecF sequences formed individual clades with MLBP/BPP of 69%/0.99 and 99%/1.0, respectively (Figure 1A). The SMC clade comprised two sub-clades, one including bacterial SMC sequences, the canonical version of archaeal SMC sequences, and SMC1-4 in eukaryotes, and the other including SMC5 and SMC6 in eukaryotes and “SMC5/6-related” version of archaeal SMC sequences. The former and latter sub-clades received MLBP/BPP of 69%/0.99 and 100%/0.99, respectively. The Arcadin-4 sequences grouped together with full statistical support and this clade was nested within the radiation of the Rad50 sequences (Figure 1A). The clade comprising the Rad50 and Arcadin-4 sequences received only an MLBP of 50% and a BPP of 0.75 (node A; Figure 1B). As the Arcadin-4 sequences are more divergent than the Rad50 sequences considered here (Figure 1A), the evolutionary relationship between Rad50 and Arcadin-4 may have been difficult to recover with confidence. Although some uncertainty remains, the SMC-superfamily phylogeny prompts us to propose the Rad50 origin of Arcadin-4. After the exclusion of the Arcadin-4 sequences, the MLBP and BPP for the monophyly of the Rad50 sequences were 73% and 0.85, respectively (Figure 1B). The presence/absence of the Arcadin-4 sequences in the alignment did not change largely the statistical supports for nodes B-D in the SMC-superfamily phylogeny (Figure 1B). The clade of the RecN sequences received MLBPs of <50% and BPPs of <0.50 and then connected with the MukB clade with MLBPs of 100% and BPPs of 1.0 (Figure 1A). Although the union of RecN and MukB was supported by high statistical support, we need to be cautious whether the two bacterial SMC-related proteins share the most recent ancestry. In particular, MukB appeared to be extremely divergent from other SMC-related proteins at the amino acid sequence level and may have introduced severe systematic artifacts in tree reconstruction (e.g., LBA).

Regardless of the presence/absence of Arcadin-4, *Sis*ClsN, *Hsa*Sph1, *Hsa*Sph2, and the archaeal proteins related to ClsN/Sph identified in this study formed a clade with full statistical support, excluding the rest of the sequences considered in the alignment (Figure 1A). This previously undescribed clade of the SMC superfamily suggests that *Sis*ClsN, *Hsa*Sph1, *Hsa*Sph2, and the archaeal proteins related to ClsN/Sph can be traced back to a single ancestral protein that is distinct from SMC, Rad50/Arcadin-4, RecF, RecN, or MukB. Takemata et al. (2019) briefly mentioned the “distant homologs of ClsN” in the species belonging to Halobacteria, Methanosarcinales, Heimdallarchaeota, and Archeoglobales. Although no detail was provided, the “distant ClsN homologs” mentioned in the pioneering study are most likely a subset of the archaeal proteins related to ClsN/Sph identified in this study. We found only three sequences related to ClsN/Sph in the bacterial metagenomes by surveying in the GenBank nr database. There are two possibilities for the three “bacterial” sequences related to ClsN/Sph. First, the three “bacterial” sequences are in fact the archaeal sequences contaminated in bacterial metagenome assemblies. Alternatively, the above-mentioned bacterial sequences may be the rare cases of lateral transfer of the archaeal gene encoded the SMC-related protein to bacterial genomes. Unfortunately, the bacterial metagenome assemblies harboring the Cls/Sph candidates range from 3.9 to 14.9 Kbp and are not sufficient to favor one of the two possibilities over the other (Supplementary Figure 2). Despite the uncertainties discussed above, the sequences related to ClsN/Sph may be, in principle, regarded as Archaea-specific. Henceforth here, we will refer to *Sis*ClsN, *Hsa*Sph1, *Hsa*Sph2, and the archaeal proteins related to ClsN/Sph as a whole as “Archaea-specific SMC-related proteins” or “ASRPs.”

**FIGURE 2:**
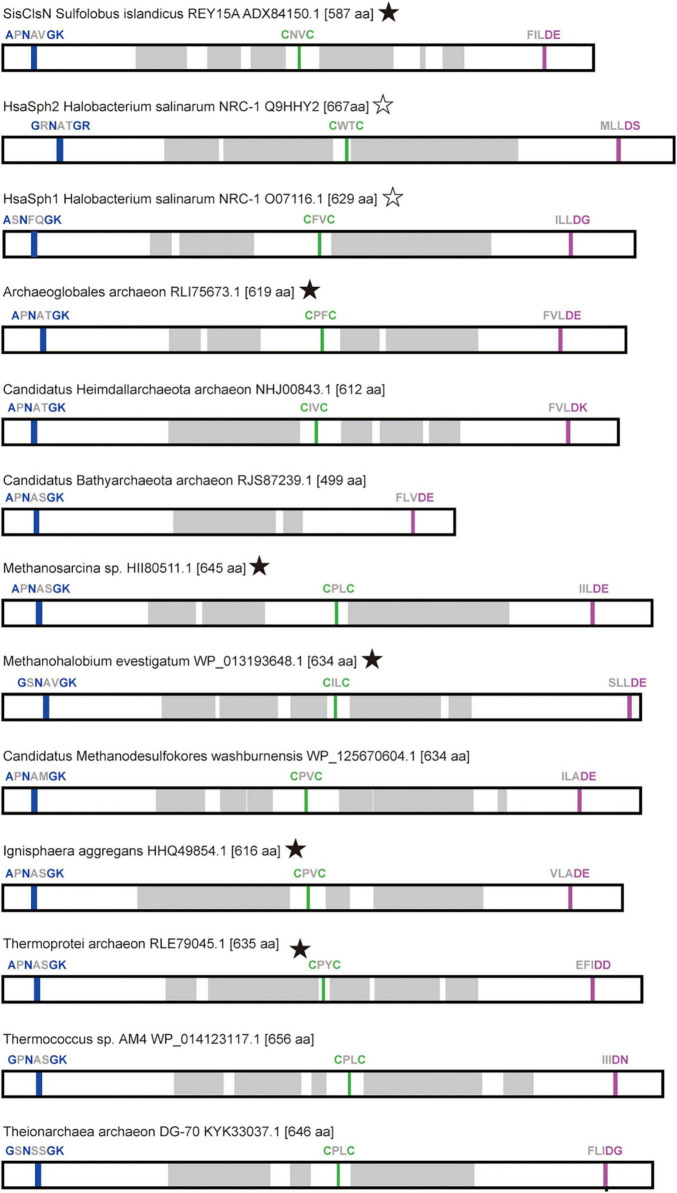
Distribution of conserved sequence motifs in the primary structures of ASRPs. For each ASRP, the distributions of Walker A, Walker B, and zinc hook motifs are shown in blue, magenta, and green, respectively. The coiled coil regions (shown in gray) were predicted by Waggawagga [http://waggawagga.motorprotein.de/; Simm et al. (2015)]. The ASPR sequences, of which tertiary structures were predicted (see Figure 3), are marked by filled stars. We marked *Hsa*Sph1 and *Hsa*Sph2 with open stars, as their predicted tertiary structures are available in AlphaFold Protein Structure Database (https://alphafold.ebi.ac.uk/).

**FIGURE 3:**
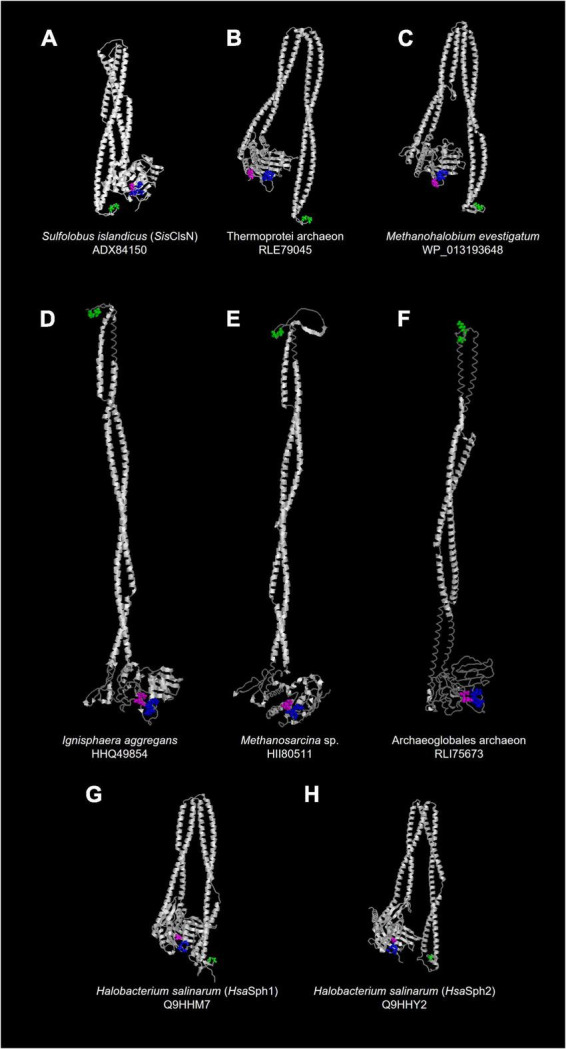
Predicted tertiary structures of ASRPs. **(A–F)** We predicted the tertiary structures of six ASRPs by AlphaFold2 (Jumper et al., 2021). The output files in the PDB format are available as Supplementary Materials. **(G,H)** The predicted tertiary structures of *Hsa*Sph1 (Q9HHM7) and *Hsa*Sph2 (Q9HHY2) were visualized from the files downloaded from AlphaFold Protein Structure Database (https://alphafold.ebi.ac.uk/). The amino acid residues in the zinc hook, Walker A, and Walker B motifs are shown in a ball-and-stick representation and colored in green, blue, and magenta, respectively.

As anticipated as SMC-related proteins, ASRPs were predicted to have the coiled-coil region, and Walker A and Walker B motifs localize at the N- and C-termini, respectively (Figure 2). Takemata et al. (2019) found a zinc hook motif, which likely facilitates the dimerization of the proteins, in the hinge region of *Sulfolobus* ClsN. This motif was found in the vast majority of the ASRP sequences analyzed in this study (including *Hsa*Sph1 and *Hsa*Sph2; Figure 2). Thus, together with Rad50, ASRP is an SMC-related protein family with a zinc hook motif. The predicted tertiary structures of *Hsa*Sph1 (Q9HHM7) and *Hsa*Sph2 (Q9HHY2) are available in AlphaFold Protein Structure Database.^1^ We predicted the tertiary structures of six additional ASRPs (including *Sis*ClsN) by AlphaFold2 (Jumper et al., 2021). Overall, the predicted tertiary structures of the ASRPs comprise the antiparallel coiled-coil and globular domain, as anticipated for SMC-related proteins (Figures 3A–F; the zinc hook, Walker A, and Walker B motifs are colored in green, blue, and magenta, respectively). The antiparallel coiled-coils in *Sis*ClsN, and two ASRPs (RLE79045 and WP_013193648) were predicted to be folded back in the middle (Figures 3A–C), resembling those in *Hsa*Sph1 and *Hsa*Sph2 (Figures 3G,H). In contrast, three predicted ASRP structures (HHQ49854, HII80511, and RLI75673) possess extended antiparallel coiled-coils (Figures 3D–F). If a pair of ASRP molecules constitutes a quaternary structure similar to the SMC complexes, the zinc hook motif and globular domain are too close in the tertiary structure with the folded antiparallel coiled coil. Thus, we anticipate that the antiparallel coiled coils in the predicted tertiary structures in Figures 3A–C,G,H are flexible and extended in their natural conformations as the predicted structures shown in Figures 3D–F.

### Neither ClsN nor Sph represents the diversity and function of ASRP

ClsN was first identified in *Sulfolobus* spp. and found to facilitate establishing the chromosome compartment with low transcriptional levels from that comprising transcriptionally active genes (Takemata et al., 2019). On the other hand, the cellular functions of Sph1 and Sph2 in *H. salinarum* have not been understood well (Herrmann and Soppa, 2002). At least there is no chromosome compartmentalization mediated by transcription in *H. salinarum* (Cockram et al., 2021), implying that the function of Sph1 or Sph2 is unlikely homologous to *Sulfolobus* ClsN. We analyzed the second alignment, which contained 44 ASRP and 28 archaeal SMC sequences (note that the latter includes both canonical and SMC5/6-related versions), to obtain the clues to examine whether the function of ASRP can be represented by ClsN or Sph1/2. In the ASRP phylogeny, *Sis*ClsN and *Hsa*Sph1/*Hsa*Sph2 were separated from each other but individually grouped with the ASRP sequences identified in the archaea closely related to *Sulfolobus* and *Halobacterium*, respectively (see below for the details). No apparent consensus in the repertory of the genes flanking an ASRP gene across genomes was found (Supplementary Table 1).

**FIGURE 4:**
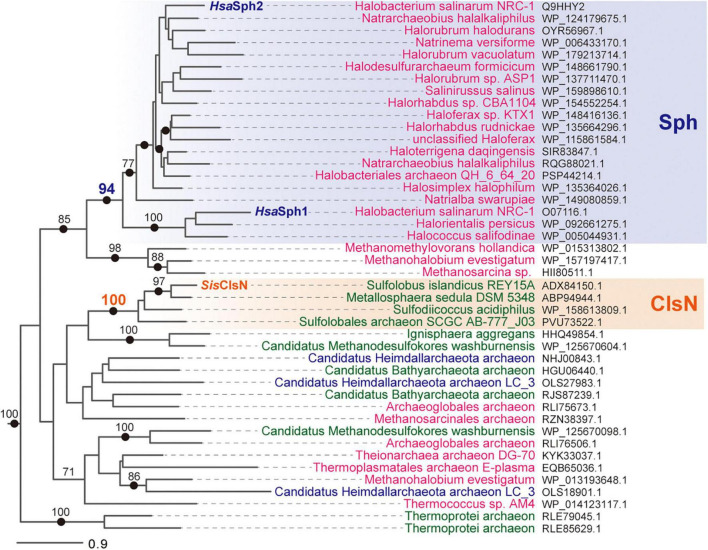
Phylogenetic relationship among 44 ASRP sequences including *Sis*ClsN, *Hsa*Sph1, and *Hsa*Sph2. The second alignment, which contained 44 ASRP and 28 archaeal SMC sequences, was subjected to both maximum-likelihood (ML) and Bayesian phylogenetic analyses. There was no substantial incongruity between the ML and Bayesian trees, and thus only the ML tree is shown. Only the clade of the ASRP sequences was presented. Non-parametric ML bootstrap support values greater than 70% and Bayesian posterior probabilities greater than 0.95 (indicated by closed dots) were shown on the corresponding nodes. The ASRP sequences of euryarchaea, crenarchaea, and Asgard archaea are colored in magenta, green, and dark blue, respectively. The Sph and ClsN clades are shaded in blue and orange, respectively.

*Sis*ClsN and the ASRP sequences of the three members of the order Sulfolobales grouped together with an MLBP of 100% and a BPP of 1.0 (Figure 4). Furthermore, three additional members of Sulfolobales were found to possess ASRP bearing an intimate phylogenetic affinity to *Sis*ClsN (Supplementary Figure 3). Altogether, the Sulfolobales ASRPs identified in this study are likely homologous to *Sulfolobus* ClsN termed as the ClsN clade in Figure 4 and Supplementary Figure 3. In the 7 members of Sulfolobales, in which ClsN sequences were detected, Rad50 sequences were constantly found albeit no SMC sequence was detected (Supplementary Figure 3). Thus, we propose that the loss of SMC and the emergence of ClsN coincided with one another and occurred in the common ancestor of the extant archaea belonging to Sulfolobales.

All of the ASRP sequences of the members belonging to the class Halobacteria grouped together with an MLBP of 94% and a BPP of 0.99 (Figure 3). *Hsa*Sph1 and *Hsa*Sph2 were distantly related to each other in this clade (Figure 2). *Hsa*Sph1 and the ASRP sequences of *Halorientalis persicus* and *Halococcus salifodinae* formed a clade with full statistical support, while the rest of the ASRP sequences of Halobacteria (including *Hsa*Sph2) grouped together with an MLBP of 77% and a BPP of 0.95 (Figure 3). Among the 80 ASRP sequences identified in this study, there were 34 ASRP sequences that were of Halobacteria but only a subset of them was included in the second alignment. Importantly, all the ASRP sequences of Halobacteria including *Hsa*Sph1 and *Hsa*Sph2 formed a highly supported clade (Supplementary Figure 3). Thus, we regard that the ASRPs of Halobacteria are functionally homologous to *Hsa*Sph1 and *Hsa*Sph2 and term the clade of these ASRP sequences as the Sph clade in Figure 3 and Supplementary Figure 3. We detected multiple distinct Sph sequences in three members of Halobacteria, namely *H. salinarum* NRC-1, *Natrarchaeobius halalkaliphilus*, and *Halobellus captivus* (marked by closed circles; Supplementary Figure 3). In contrast to the Sulfolobales archaea in which no co-occurrence of ClsN and SMC was observed, most of the members of Halobacteria appeared to possess both SMC and Rad50, as well as Sph. The co-occurrence of SMC and Sph implies a certain level of difference in function between the two proteins.

The three ASRP sequences of *Methanomethylovorans hollandica*, *Methanohalobium evestigatum*, and *Methanosarcina* sp. formed a well-supported clade, which was connected to the Sph clade with an MLBP of 85% and a BPP of 0.97 (Figure 4). Despite the phylogenetic affinity to the Sph clade, we do not regard the three ASRP sequences as the Sph homologs, as they were identified from the species that do not belong to Halobacteria. Otherwise, any ASRP sequence identified in the members of the TACK superphylum, Euryarchaeota, and Asgard group showed no apparent phylogenetic affinity to the ClsN or Sph clade (Figure 4). Multiple distinct ASRP sequences were found in each of *M. evestigatum*, *Candidatus* Methanodesulfokores washburnensis, and *Candidatus* Heimdallarchaeota archaeon LC 3 (marked by closed circles; Supplementary Figure 2). The ASRP sequences, which were excluded from both Sph and ClsN clades, co-occur with both SMC and Rad50 sequences in the corresponding genomes. Thus, if ASRP and SMC co-exist, we anticipate that the functions of the two proteins are distinct from one another.

## Conclusion

We here report the novel SMC-related protein family found exclusively in Archaea. Both of the two SMC-related proteins identified previously in *Sulfolobus* spp. and *H. salinarum*, namely ClsN and Sph, belong to this protein family that was termed ASRP here. The original function of ASRP currently remains uncertain but is likely distinctive from that of ClsN or Sph. If so, the function of ASRP may have been remodeled in the common ancestor of Sulfolobales and that of Halobacteria separately and uniquely. Our proposals above stand solely on the phylogenetic distribution and thus need to be considered tentative. To estimate the function of the ancestral ASRP and retrace the changes in the ASRP function, the cellular functions of ASRPs of phylogenetically broad archaea including Sph are indispensable as the most fundamental information.

## Data Availability

The original contributions presented in this study are included in the article/Supplementary material, further inquiries can be directed to the corresponding author.

## Footnotes

1
https://alphafold.ebi.ac.uk/
