# Supplementary material for: A novel structural maintenance of chromosomes (SMC)-related protein family specific to Archaea
Source: Front Microbiol. 2022 Aug 5;13:913088. doi: 10.3389/fmicb.2022.913088 (PMC9389158; doi:10.3389/fmicb.2022.913088)
Supplement: Supplementary file 3 [file Data_Sheet_3.PDF]

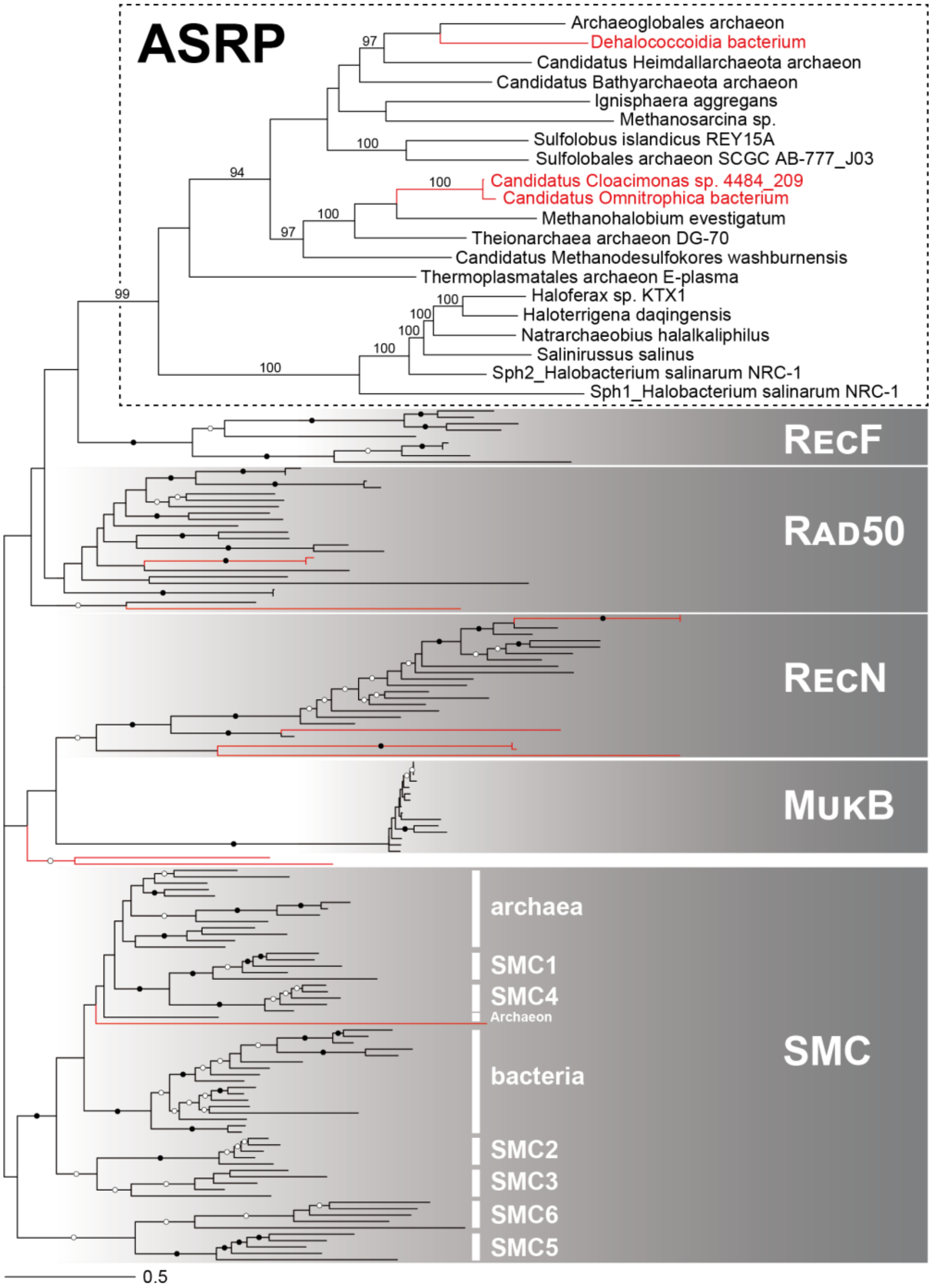

Supplementary Figure 1. Maximum-likelihood analysis of ClsN/Sph candidates found in bacterial genomes/metagenomes. The branches correspond to the bacterial ClsN/Sph candidates are highlighted as red. Only three sequences formed a clade with the ClsN/Sph candidates found in the archaeal genomes/metagenomes. The ML tree was inferred with the LG + Γ + F model. Ultrafast bootstrap support values (UFBPs) equal to or greater than 90% were shown on the corresponding nodes. Closed and open dots indicate UFBPs of 100% and UFBPs equal to or greater than 90%, respectively.

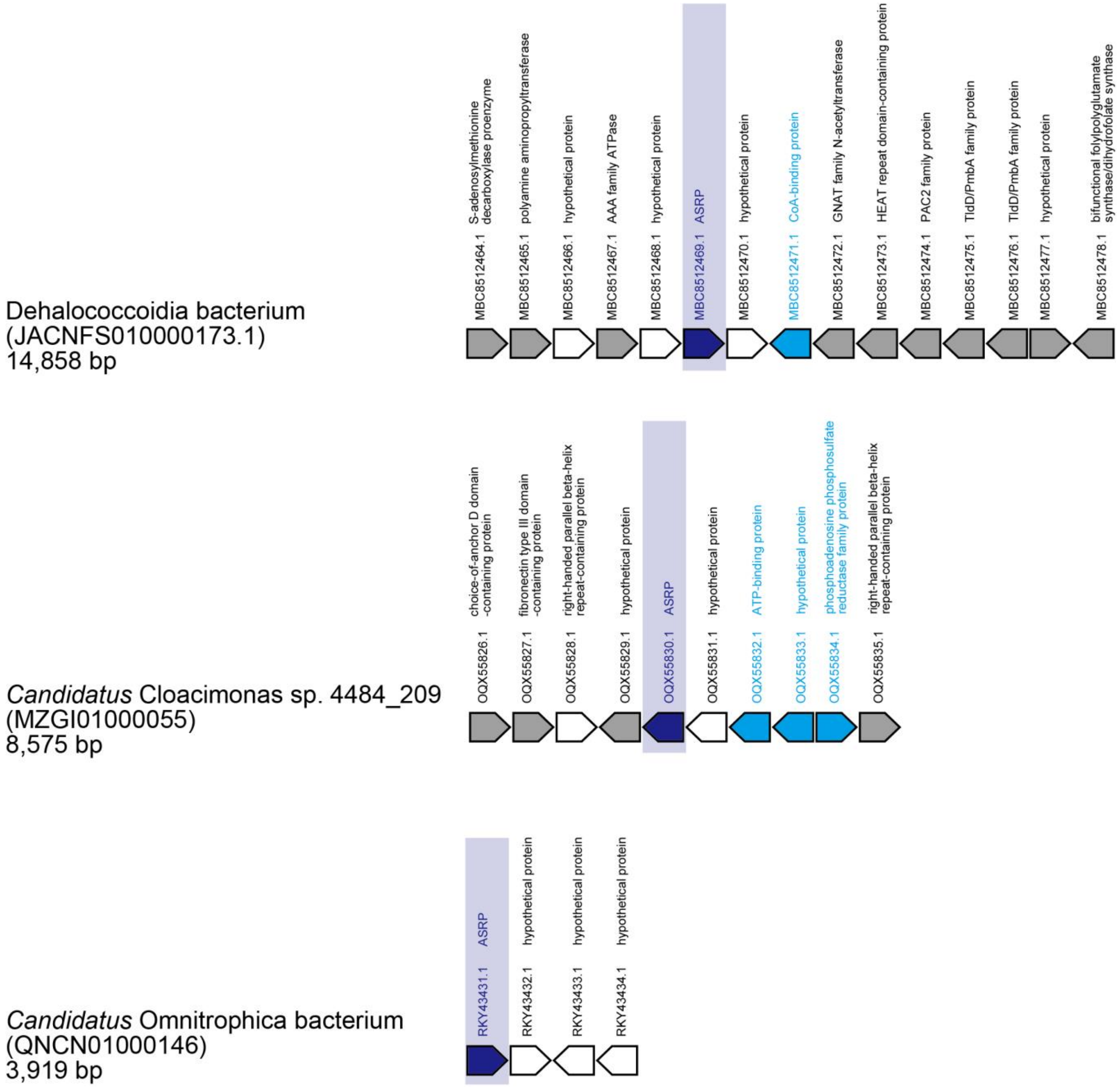

Supplementary Figure 2. Genes surrounding 'ASRP' genes in three bacterial metagenome assemblies. Right- and left-pointing boxes represent the genes on the sense and antisense strands, respectively. The conceptual amino acid sequences were individually subjected to BLASTP against GenBank RefSeq Selected proteins. Grey and blue boxes represent the genes with the sequence homology to bacterial and archaeal proteins, respectively. Open boxes indicate the genes showed no significant homology to any sequences in the database.

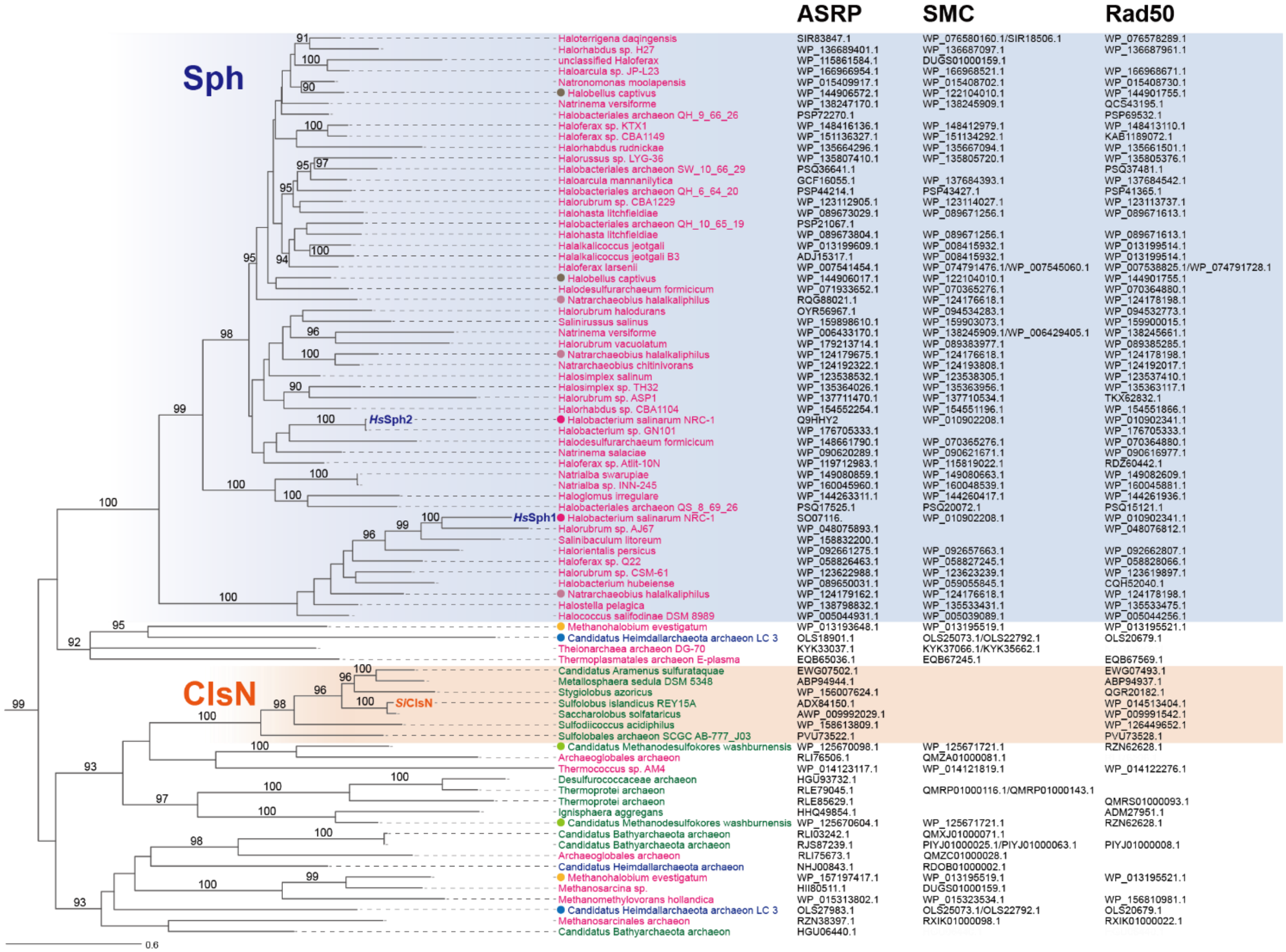

Supplementary Figure 3. Maximum-likelihood analysis of 83 ASRP sequences. The ML tree was inferred with the LG + Γ + F model. Ultrafast bootstrap support values (UFBPs) equal to or greater than 90% were shown on the corresponding nodes. The details of this figure are the same as Fig. 2. The ASRP sequences, which were detected in the same genome, are marked by dots. The difference in the color of dots displays the difference in genome/metagenome. For each genome/metagnome in which an ASRP sequence was found, we searched for SMC and Rad50 sequences and listed their GenBank accession numbers. In case of no SMC/Rad50 sequence being not found, we left the corresponding space empty.

Table S1. Genes neighboring an ASRP gene

Organism (Euryarchaeota/ Crenarchaeota/Asgard group)	GenBank Accession no. of the genomic data	-3 ORF	-2 ORF	-1 ORF	ASRP	+1 ORF	+2 ORF	+3 ORF
Metallosphaera sedula DSM 5348	ABP94944.1	CBS domain containing protein	ferric uptake regulator, Fur family	Inosine/uridine-preferring nucleoside hydrolase	ABP94944.1	hypothetical	CBS domain containing protein	metallophosphoesterase
Halalkalicoccus jeotgali B3	CP002062	ferredoxin	Alcohol dehydrogenase GroES domain protein	Transposase and inactivated derivatives-like protein	ADJ15317.1	hypothetical	galactonate dehydratase	Glyoxalase/bleomycin resistance protein/dioxygenase
Sulfolobus islandicus REY15A	CP002425	phosphoglycerate mutase	metallophosphoesterase	hypothetical	ADX84150.1	Inosine/uridine-preferring nucleoside hydrolase	ferric uptake regulator, Fur family	hypothetical
Thermoplasmatales archaeon E-plasma	ATME01000013.1	hypothetical	sugar transporter	hypothetical	EQB65036.1	hypothetical	hypothetical	AAA ATPase, CDC48
Candidatus Aramenus sulfurataquae	ASRH01000003.1	hypothetical	Fur family ferric uptake regulator	inosine/uridine-preferring nucleoside hydrolase	EWG07502.1	hypothetical	signal-transduction protein	metallophosphoesterase
Haloarcula mannanilytica	BIXZ01000014.1	acetolactate synthase	LLM class F420-dependent oxidoreductase	IclR family transcriptional regulator	GCF16055.1	hypothetical	chromosome partitioning protein ParA	hypothetical
Candidatus Bathyarchaeota archaeon	DTEA01000543.1	hypothetical	AAA family ATPase	hypothetical	HGU06440.1	AbrB/MazE/SpoVT family DNA-binding domain-containing protein	recombinase	no data
Desulfurococcaceae archaeon	DTDO01000044.1	hypothetical	hypothetical	AAA family ATPase	HGU93732.1	no data	no data	no data
Ignisphaera aggregans	DRYQ01000009.1	hypothetical	hypothetical	hypothetical	HHQ49854.1	hypothetical	AAA family ATPase	hypothetical
Methanosarcina sp.	DUGS01000138.1	hypothetical	hypothetical	hypothetical	HII80511.1	ATP-binding protein	hypothetical	hypothetical
Methanomethylovorans hollandica	CP003363	hypothetical	hypothetical	hypothetical	WP_015313802.1	hypothetical	trypsin-like serine protease with C-terminal PDZ domain	hypothetical
Theionarchaea archaeon DG-70	LSSB01000151.1	hypothetical	hypothetical	hypothetical	KYK33037.1	hypothetical	hypothetical	hypothetical
Candidatus Heimdallarchaeota archaeon	RDOB01000002.1	DUF504 domain-containing protein	TatD family deoxyribonuclease	hypothetical	NHJ00843.1	hypothetical	DNA-binding protein	hypothetical
Halobacterium salinarum NRC-1	CP038632	IclR family transcription regulator ArcR	hypothetical	hypothetical	O07116.1	XerC/D-like integrase	hypothetical	hypothetical
Halobacterium salinarum NRC-1	BK010831.1	IS1341-type transposase /UspA domain protein	spurious ORF	hypothetical	Q9HHY2	TrkA domain protein	"potassium-transporting ATPase subunit F	potassium-transporting ATPase subunit A
Candidatus Heimdallarchaeota archaeon LC_3	MDVS01000131.1	hypothetical	hypothetical	hypothetical	OLS18901.1	hypothetical	Proteasome-associated ATPase	hypothetical
Candidatus Heimdallarchaeota archaeon LC_3	MDVS01000131.1	VCP-like ATPase	hypothetical	hypothetical	OLS27983.1	hypothetical	Nucleoid occlusion protein	hypothetical
Halorubrum halodurans	NHPJ01000072.1	hypothetical	hypothetical	hypothetical	OYR56967.1	cell division control protein Cdc6	hypothetical	hypothetical
Halobacteriales archaeon QH_10_65_19	PXQK01000150.1	heme ABC transporter ATP-binding protein	ribose ABC transporter permease	ABC transporter permease	PSP21067.1	hypothetical	CBS domain-containing protein	initiation factor 2B
Halobacteriales archaeon QH_6_64_20	PXQR01000022.1	hypothetical	UDP-glucose 4-epimerase	BCCT family transporter	PSP44214.1	hypothetical	sugar ABC transporter ATP-binding protein	alpha-xylosidase
Halobacteriales archaeon QH_9_66_26	PXRD01000047.1	coenzyme A pyrophosphatase	hypothetical	restriction endonuclease	PSP72270.1	hypothetical	oxidoreductase	5-carboxymethyl-2-hydroxymuconate isomerase
Halobacteriales archaeon QS_8_69_26	PXRX01000023.1	3-isopropylmalate dehydratase large subunit	3-isopropylmalate dehydratase small subunit	NAD-dependent isocitrate dehydrogenase	PSQ17525.1	hypothetical	AsnC family protein	hypothetical

Organism (Euryarchaota/ Crenarchaeota/Asgard group)	GenBank Accession no. of the genomic data	-3 ORF	-2 ORF	-1 ORF	ASRP	+1 ORF	+2 ORF	+3 ORF
Halobacteriales archaeon SW_10_66_29	PXSF01000070.1	DNA topoisomerase I	transposase	aryl-alcohol dehydrogenase	PSQ36641.1	hypothetical	xylulose kinase	IclR family transcriptional regulator
Sulfolobales archaeon SCGC AB-777_J03	QEFM01000002.1	phosphoglycerate mutase	CBS domain-containing protein	hypothetical	PVU73522.1	nucleoside hydrolase	transcriptional repressor	hypothetical
Candidatus Bathyarchaeota archaeon	PIYJ01000034	NAD-dependent epimerase/dehydratase family protein	glucose-1-phosphate thymidylyltransferase	hypothetical	RJS87239.1	hypothetical	hypothetical	hypothetical
Thermoprotei archaeon	QMRP01000187.1	no data	no data	hypothetical	RLE79045.1	hypothetical	no data	no data
Thermoprotei archaeon	QMRS01000181.1	hypothetical	hypothetical	hypothetical	RLE85629.1	hypothetical	no data	no data
Candidatus Bathyarchaeota archaeon	QMXJ01000104.1	hypothetical	hypothetical	hypothetical	RLI03242.1	no data	no data	no data
Archaeoglobales archaeon	QMZC01000134.1	hypothetical	hypothetical	hypothetical	RLI75673.1	no data	no data	no data
Archaeoglobales archaeon	QMZA01000005.1	NAD-dependent protein deacylase	DUF554 domain-containing protein	hypothetical	RLI76506.1	hypothetical	hypothetical	hypothetical
Natrarchaeobius halalkaliphilus	REFY01000005.1	hypothetical	methyltransferase domain-containing protein	DUF4255 domain-containing protein	RQG88021.1	hypothetical	PAS domain S-box protein	response regulator
Natrarchaeobius halalkaliphilus	REFY01000005.2	ABC transporter permease	ABC transporter permease	ABC transporter substrate-binding protein	WP_124179162.1	hypothetical	ParA family protein	PAS domain-containing protein
Natrarchaeobius halalkaliphilus	NZ_REFY01000007	orc1/cdc6 family replication initiation protein	acetamidase/formamidase family protein	AmiS/UreI family transporter	WP_124179675.1	hypothetical	AMP-binding protein	TCP-1/cpn60 chaperonin family protein
Methanosarcinales archaeon	RXIK01000079.1	hypothetical	hypothetical	hypothetical	RZN38397.1	hypothetical	ATP-binding protein	end of the assembly
Haloterrigena daqingensis	FTNP01000003.1	Acyl dehydratase	hypothetical	hypothetical	SIR83847.1	hypothetical protein	transcriptional regulator, IclR family	Acyl-CoA dehydrogenase
Halococcus salifodinae	NZ_AOME01000075	7,8-didemethyl-8-hydroxy-5-deazariboflavin synthase subunit CofH	nucleotidyltransferase family protein	hypothetical	WP_005044931.1	acetolactate synthase large subunit	acetolactate synthase large subunit	NCS2 family permease
Natrinema versiforme	NZ_AOID01000064	formyltetrahydrofolate deformylase	DHHA1 domain-containing protein	dihydropteroate synthase	WP_005044931.1	hypothetical	helix-turn-helix domain-containing protein	hypothetical
Haloferax larsenii	NZ_AOLI01000015	APC family permease	universal stress protein	hypothetical	WP_007541454.1	type 1 glutamine amidotransferase	SprT-like domain-containing protein	ATP-binding protein
Saccharolobus solfataricus	NZ_CP033238.1	2,3-diphosphoglycerate-dependent phosphoglycerate mutase	metallophosphoesterase	hypothetical	WP_009992029.1	IS6-like element ISC774 family transposase	IS256 family transposase	nucleoside hydrolase
Methanohalobium evestigatum	NC_014253	hypothetical	ATP-binding protein	hypothetical	WP_013193648.1	hypothetical	phosphoadenosine phosphosulfate reductase family protein	hypothetical
Methanohalobium evestigatum	NC_014254	fibronectin type III domain-containing protein	PGF-pre-PGF domain-containing protein	surface glycoprotein	WP_157197417.1	hypothetical	hypothetical	IS200/IS605 family element RNA-guided endonuclease TnpB
Halalkalicoccus jeotgali	NC_014298	Halalkalicoccus jeotgali	Halalkalicoccus jeotgali	hypothetical	WP_013199609.1	orc1/cdc6 family replication initiation protein	hypothetical	hypothetical

Organism (Euryarchaota/ Crenarchaeota/Asgard group)	GenBank Accession no. of the genomic data	-3 ORF	-2 ORF	-1 ORF	ASRP	+1 ORF	+2 ORF	+3 ORF
Thermococcus sp. AM4	NC_016051	hypothetical	hypothetical	hypothetical	WP_014123117.1	hypothetical	ATP-binding protein	hypothetical
Natronomonas moolapensis	NC_020388	endonuclease III	hypothetical	hypothetical	WP_015409917.1	SDR family NAD(P)-dependent oxidoreductase	phytoene desaturase family protein	prenyltransferase
Halorubrum sp. AJ67	NZ_CBVY010000001.1	hypothetical	hypothetical	hypothetical	WP_048075893.1	hypothetical	hypothetical	GAF domain-containing protein
Haloferax sp. Q22	NZ_LOEP01000014.1	hypothetical	hypothetical	hypothetical	WP_048075893.1	hypothetical	V-type ATP synthase subunit D	amino acid permease
Halodesulfurarchaeum formicicum	NZ_CP016804.1	MFS transporter	DUF1638 domain-containing protein	IclR family transcriptional regulator	WP_071933652.1	hypothetical	hypothetical	amidohydrolase
Halobacterium hubeiense	NZ_CEMR01000011.1	hypothetical	tautomerase	HD domain-containing protein	WP_089650031.1	hypothetical	helix-turn-helix domain-containing protein	helix-turn-helix domain-containing protein
Halohasta litchfieldiae	NZ_AOME01000075	7,8-didemethyl-8-hydroxy-5-deazariboflavin synthase subunit CofH	nucleotidyltransferase family protein	hypothetical	WP_089673029.1	acetolactate synthase large subunit	NCS2 family permease	NADPH-dependent F420 reductase
Halohasta litchfieldiae	FNYR01000046	hypothetical	hypothetical	hypothetical	WP_089673804.1	TspO and MBR related proteins	glucose-1-phosphate thymidylyltransferase	UDPglucose 6-dehydrogenase
Natrinema salaciae	NZ_FOFD01000005.1	hypothetical	orc1/cdc6 family replication initiation protein	orc1/cdc6 family replication initiation protein	WP_090620289.1	hypothetical	IclR family transcriptional regulator	SDR family oxidoreductase
Halorientalis persicus	NZ_FOCX01000013.1	MaoC family dehydratase	AMP-binding protein	CoA ester lyase	WP_092661275.1	hypothetical	hypothetical	hypothetical
Haloferax sp. Atlit-10N	PSYX01000016.1	hypothetical	hypothetical	archaeosortase A	WP_119712983.1	hypothetical	transposase	spermidine synthase
Halorubrum sp. CBA1229	NZ_CP054585.1	thiamine pyrophosphate-dependent dehydrogenase E1 component subunit alpha	hypothetical	hypothetical	WP_123112905.1	DUF362 domain-containing protein	IclR family transcriptional regulator	MFS transporter
Halosimplex salinum	NZ_QWGG01000008.1	sulfite exporter TauE/SafE family protein	DUF4112 domain-containing protein	helix-turn-helix domain-containing protein	WP_123538532.1	DUF2196 domain-containing protein	CBS domain-containing protein	hypothetical
Halorubrum sp. CSM-61	NZ_RJJW01000007.1	cytochrome P450	hypothetical	hypothetical	WP_123622988.1	hypothetical	hypothetical	hypothetical
Natrarchaeobius chitinivorans	NZ_REFZ01000010.1	thermosome subunit 1	AMP-binding protein	hypothetical	WP_123622988.1	AmiS/UreI transporter	acetamidase/formamidase family protein	"orc1/cdc6 family replication initiation protein
Candidatus Methanodesulfokores washburnensis	RCOS01000011	hypothetical	DGQHR domain-containing protein	hypothetical	WP_125670098.1	no data	no data	no data
Candidatus Methanodesulfokores washburnensis	RXII01000078	hypothetical	AAA family ATPase	hypothetical	WP_125670604.1	no data	no data	no data
Halosimplex halophilum	NZ_ML214297.1	LEA type 2 family protein	cupin domain-containing protein	hypothetical	WP_135364026.1	hypothetical	alpha-D-ribose 1-methylphosphonate 5-triphosphate diphosphatase	hypothetical
Halorhabdus rudnickae	NZ_CAAHFB01000000	hypothetical	hypothetical	hypothetical	WP_135664296.1	hypothetical	IS6 family transposase	hypothetical

Organism (Euryarchaota/ Crenarchaeota/Asgard group)	GenBank Accession no. of the genomic data	-3 ORF	-2 ORF	-1 ORF	ASRP	+1 ORF	+2 ORF	+3 ORF
Halorussus sp. LYG-36	NZ_SBIV01000018.1	aldolase/citrate lyase family protein	RidA family protein	IclR family transcriptional regulator	WP_135807410.1	hypothetical	IS66 family transposase	hypothetical
Halorubrum sp. ASP1	SGUA01000007.1	DNA adenine methylase	hypothetical	hypothetical	WP_137711470.1	hypothetical	hypothetical	hypothetical
Natrinema versiforme	NZ_CP040331.1	UbiX family flavin prenyltransferase	TRAP transporter fused permease subunit	amidohydrolase	WP_138247170.1	hypothetical	benzoate/H(+) symporter BenE family transporter	DUF6282 family protein
Halostella pelagica	NZ_SJES01000012.1	AsnC family transcriptional regulator	hypothetical	hypothetical	WP_138798832.1	hypothetical	amidohydrolase family protein	amidohydrolase family protein
Haloglomus irregulare	NZ_QMDX01000017.1	PD-(D/E)XK nuclease family protein	carboxymuconolactone decarboxylase family protein	hypothetical	WP_144263311.1	amidohydrolase family protein	iron-sulfur cluster assembly protein	AMP-binding protein
Halobellus captivus	NZ_VJXQ01000005.1	IS6 family transposase	IS5 family transposase	hypothetical	WP_144906017.1	IS6 family transposase	DDE-type integrase/transposase/recombinase	IclR family transcriptional regulator
Halobellus captivus	NZ_VJXQ01000007	IclR family transcriptional regulator	IS5/IS1182 family transposase	hypothetical	WP_144906572.1	IS6 family transposase	hypothetical	Fic family protein
Halodesulfurarchaeum formicicum	NZ_CP016804.1	hypothetical	hypothetical	DUF1059 domain-containing protein	WP_148661790.1	hypothetical	IclR family transcriptional regulator	aldehyde dehydrogenase family protein
Natrialba swarupiae	VTAW01000007.1	hydantoinase/oxoprolinase family protein	AsnC family transcriptional regulator	hypothetical	WP_149080859.1	aldehyde dehydrogenase family protein	ABC transporter substrate-binding protei	allantoinase AllB
Haloferax sp. CBA1149	VZUS01000001.1	DUF4129 domain-containing protein	DUF1616 domain-containing protein	hypothetical	WP_151136327.1	hypothetical	MoaD/ThiS family protein	hypothetical
Stygiolobus azoricus	NZ_CP045483.1	2,3-diphosphoglycerate-dependent phosphoglycerate mutase	CBS domain-containing protein	hypothetical	WP_156007624.1	nucleoside hydrolase	transcriptional repressor	hypothetical
Sulfodiicoccus acidiphilus	NZ_BMQS01000012	iron-containing alcohol dehydrogenase	thiamine pyrophosphate-binding protein	SMP-30/gluconolactonase/LRE family protein	WP_158613809.1	nucleoside hydrolase	transcriptional repressor	hypothetical
Natrialba sp. INN-245	WUBV01000005.1	hydantoinase/oxoprolinase family protein	AsnC family transcriptional regulator	hypothetical	WP_160045960.1	aldehyde dehydrogenase family protein	hypothetical	allantoinase AllB
Haloarcula sp. JP-L23	NZ_CP050014.1	winged helix-turn-helix domain-containing protein	hydrogenase maturation nickel metallochaperone HypA	lipoyl synthase	WP_166966954.1	hypothetical	HAMP domain-containing histidine kinase	hypothetical
Halorubrum vacuolatum	NZ_FZNQ01000048.1	no data	no data	hypothetical	WP_179213714.1	response regulator	response regulator	hypothetical
